# Epidemiological Study of *Toxocar canis* in Children under 14-Years-Old and Dogs in Zabol and Chabahar Districts, Southeast of Iran

**Published:** 2017

**Authors:** Mehdi KHOSHSIMA SHAHRAKI, Mansour DABIRZADEH, Mahdi AFSHARI, Yahya MAROUFI

**Affiliations:** 1. Dept. of Parasitology & Mycology, Zabol University of Medical Sciences, Zabol, Iran; 2. Dept. of Community Medicine, Zabol University of Medical Sciences, Zabol, Iran

**Keywords:** *Toxocara canis*, Sero-epidemiology, Dogs, Children, Iran

## Abstract

**Background:**

The purpose of this study was seroepidemiological and parasitological assessment of *Toxocara canis* infection in children and dogs in Zabol and Chabahar, Iran.

**Methods:**

This study was a descriptive-analytic study with a simple random sampling of children under 14 yr old, referring to urban, rural, and tribal laboratories of Zabol and Chabahar, Sistan and Baluchestan Province, Iran in 2016. Demographic data, clinical, and laboratory conditions of patients were collected through interviews, questionnaires, and blood count measuring. The prevalence of IgG antibodies against *T. canis* was assessed by ELISA. *T. canis* eggs in dogs (as the original host) were also assessed by examining animal feces. Then the data were analyzed using SPSS 19 software and descriptive statistics, chi-square and ANOVA statistical tests.

**Results:**

Totally, 364 patients were enrolled, of which 51.6% were female and mean±SD age of participants was 7.2 (±3.7) yr. IgG antibodies against *T. canis* was observed in 3.8% of cases. A significant association was found between the seroprevalence of *T. canis* and eosinophil (*P*=0.003) and red blood cell count (*P*=0.04). We also found a significant association between serological prevalence of *T. canis* and demographic parameters, such as city of residence (*P*=0.003), gender (*P*=0.04), consumption of vegetables *(P*=0.01), and the living place (*P*=0.04). Mean antibody titration was 2.2 ±1.1, with statistically significant difference among age groups (*P*=0.001). In addition, *T. canis* infection was positive in 27.5% of dogs living in the study areas.

**Conclusion:**

High risk of infection represented in patients referring to laboratories of Zabol and Chabahar. In addition, given the fact that dogs are the final hosts to transfer *Toxocara* infection to humans, this study emphasizes the need to control the population of stray dogs in the region to prevent the development of disease in the human society.

## Introduction

Toxocariasis is a parasitic disease caused by infection with a group of nematodes of the *Toxocara* genus from roundworms (1). The most common parasitic agent of toxocariasis is *T. canis* (dogs’ *Ascaris*). It is considered as common zoonotic worm infection (2). The *T. canis* eggs can hardly be cleaned from dogs’ living and defecation place. The infectious *T. canis* eggs can be swallowed by humans, as a random host and be opened consequently in the intestine and release larvae. Then, the larvae can penetrate the intestinal wall and migrate to liver, brain, eyes, and other tissues through the blood and lymph vessels. In these organs, the larvae can remain without growth and differentiation for a long time (months) as migrant larvae that are metabolically active. The larvae can cause severe local reactions (3, 4).

Toxocariasis is considered as a common parasitic infection in children. The population at risk becomes infected by ingestion of embryonated eggs from soil, dirty hands, and raw vegetables or undercooked meat containing transitional hosts’ larvae (transient), such as chicken, beef, and sheep. Human infection and the larvae can be transmitted to humans by eating flesh of the transient hosts as a paratenic host) (5). Inflammatory reactions in children present with fever, cough, wheezing due to pneumonia, and gastrointestinal symptoms including lethargy, lack of appetite, vomiting, abdominal pain, general weakness, malnutrition, vague hepatomegaly and splenomegaly, muscle aches, and joint pains (6).

Infections in dogs, especially in stray dogs, are an important epidemic factor in nature (7). According to reports, 76% of dogs can be infected with this parasite, for example, dogs’ infections were reported as of 51% in Hamadan, Iran (8). Each female worm that resides in the intestines of the infected dogs can spread 200000 eggs in human living place and, therefore, examining dogs for spread of the infection is of great importance (7, 9). There is no evidence about the prevalence of this parasite in children under 14 yr in the area.

Regarding the risk factors for transmission of the parasite such as seasonal winds in Zabol and Chabahar, the presence of eggs in the atmosphere of the city is very likely. Therefore, the favorable climatic conditions in the city (the hot and humid weather), which can cause eggs lasting in the environment, demonstrates the need for epidemiological studies on the prevalence of this parasite more than ever (7).

In this study, for the first time in Sistan and Baluchestan, Iran, the seroprevalence of toxocariasis infection among children fewer than 14 yr old in rural, urban, and tribal laboratories as well as *Toxocara* infection in dogs living in Zabol and Chabahar districts were assessed.

## Materials and Methods

Chabahar, formerly Bandar Beheshti is capital of Chah Bahar County, Sistan and Baluchestan Province, Iran. Chabahar is a free port (Free Trade Zone) on the coast of the Gulf of Oman. Chabahar is Iran’s southernmost city. The county of Chabahar has hot, humid weather in the summer and warm weather in the winter, giving it a hot desert climate. The western winds in the winter bring about scattered rainfalls in this region, and very occasionally, winds from the Indian monsoon affect the region. In the summer monsoon, winds from the Indian subcontinent make Chabahar the coolest southern port in the summer and the warmest port of Iran in the winter. It has an average maximum temperature of 34 °C and an average minimum temperature of 21.5 °C (10).

Zabol is the capital of Zabol County, Sistan and Baluchestan Province, southeast Iran. Zabol lies on the border with Afghanistan that referred to as Sistan until the late 1920s. Weather: 28 °C, Wind NW at 42 km/h, 25% Humidity. Zabol is located near Lake Hamun and Hirmand River irrigated the region. Lake Hamun is a seasonal lake that is often dry.

The Zabel area is well-known for its “120-day wind”, a highly persistent dust storm in the summer which blows from north to south (11).

This study was a descriptive-analytic study carried out with a simple random sampling among children under 14 yr referring to private, public, and tribal laboratories of Zabol and Chabahar, Iran, from 2014–2015. Demographic data was collected by laboratory personnel stationed in the laboratory and through face-to-face interview, completing checklist, and blood samples by laboratory experts. Three labs were selected in each city, considering the situation of labs, public health lab, private and hospital lab (outpatient), respectively. The sample size was calculated as of 364 children using parameters obtained based on previous studies (1). Proportional to sizes of the study districts, 124 samples were collected from Chabahar and 240 samples from Zabol.

Informed consent was taken from the participants and the study was approved by Ethics Committee of the university.

### Determination of serum antibodies level against Toxocara

To measure titration of IgG antibody against antigens of *Toxocara*, first 4 ml venous blood samples were collected from participants. Samples were divided into two parts: one part was used for blood cell count and another to measure IgG anti-*Toxocara* by ELISA method. The ELISA kit intended for testing the serum levels of IgG against *Toxocara* was purchased from IBL (Germany) and manufacturer’s protocol was used for testing.

### Assessing the prevalence of Toxocara infection in dogs of the region

The existence of *T. canis* parasite eggs in the feces of 40 dogs of the area was investigated. Twenty dog’s fecal samples were taken from Zabol and twenty samples from Chabahar. Ten samples were from villages of both Chabehar and Zabol.

Thirty cases were collected from many points of the both cities. After collecting dogs’ fecal samples with full hygienic consideration, samples were prepared using sedimentation techniques by formalin-ether method. *T. canis* parasite eggs were assessed in the sedimentation by light microscopy and lenses 10 and 40 to find the infection in dogs.

### Statistical analysis

Descriptive statistics such as mean, and frequency were used and for statistical analysis, chi square test was used. All analyses were performed using SPSS19 software (Chicago, IL, USA). *P*-value less than 0.05 was considered significant.

## Results

Out of 364 participants, 188 patients (51.6%) were female (Table. 1). The mean (standard deviation) age of participants was 3.7(7.2) yr. Overall, 240 cases (65.1%) lived in Zabol and 124 (34.9%) lived in Chabahar. 24.17% of them had history of exposure to dogs. 3.8% (14/364 cases) of anti-*Toxocara* antibodies were positive. The mean (SD) age of the positive cases was 7.9 (4) yr and the mean age of negative cases were 7.2 (±3) yr. Significant correlation was found between toxocariasis antibody and demographic, clinical, or laboratory parameters (Table 1). Mean (SD) antibody titer was 2.2(±1.1) in the studied cases. No significant differences in mean antibody titer were observed among the three age groups: 1–5, 6–10, and over 10 yr. Significant association was seen between serological prevalence of *T. canis* and laboratory finding such as eosinophilia (*P*=0.003) and red blood cells count (*P*=0.03). The results showed a significant association between the serological prevalence of *T. canis* and demographic characteristics such as city of residence (*P*=0.003), gender (*P*=0.04), consumption of vegetables (*P*=0.01), and living place (*P*=0.04). *Toxocara* eggs were found in 11 cases (27.5%) of 40 dogs’ stool. In addition, *Toxocara* eggs were found in 8 out of 30 dogs’ feces (26%) living in urban areas, and 3 out of 10 dogs in rural area (30%).

**Table 1:** Seroprevalence of *Toxocara* according to the laboratory results

**Lab results**		**Negative serology N (%)**	**Positive serology N (%)**	***P* value**
Eosinophil (%)	0–5	133(100)	0(0)	0.003
	>6	217(93/9)	14(6/1)	
Hemoglobin	Normal	239(96/8)	8(3/2)	0.4
	Low	111(94/9)	6(5/1)	
WBC count	Normal	287(96)	12(4)	0.7
	High	63(96/9)	2(3/1)	
RBC count	Normal	295(97)	9(3)	0.04
	Low	55(91/7)	5(8/3)	
Platelet	Normal	319(96/7)	11(3/3)	0.1
	Low	31(91/2)	3(8/8)	

## Discussion

In this study, for the first time, human IgG antibody titer of toxocariasis or migrant visceral larva were assessed in children under 14 yr referring to laboratories of University of Medical Sciences of Chabahar, Zabol in Iran, and the villages of these two cities. 3.8% of these children have experienced *Toxocara* infections. The prevalence of toxocariasis in children under 14 yr had significant correlation with the place of residence, gender, consumption of vegetables per week, nomadic life, and laboratory characteristics, such as high eosinophils and anemia.

In this study, seroprevalence of *T. canis* was totally calculated as 3.8%, which was 1.7% in Zabol, 1.8% in Chabahar, and 3.31% among local tribes. Our results were similar to results reported from Hamedan, less than Kermanshah, Tabriz, Shiraz, and more than Zanjan in Iran (Table 1) (1, 12). The basis of the prevalence difference among different cities could be related to different climates and cultural habits of the studied populations. Moreover, the similarity of results of these two districts can be due to similarity in cultures of these two areas such as keeping dogs and food habits (13). The higher titer of *T. canis* IgG in children fewer than 14 in Chabahar compared to Zabol can also be related to different climates, cultural habits, and high humidity. The lower statistics of the current study compared to other studies conducted in other countries can be due to difference in cultural habits, method of cooking food, climates and soil condition, the plants distribution, and humidity level. In addition, the higher prevalence of *Toxocara* in the mentioned countries might be due to larger age range of these participants and stability of larvae until 10 yr in human body in older patients (7, 13). Although studies that are more extensive are required to assess the infection in the province, researchers have associated the reason of this difference in different areas with factors such as climate, soil, and plants distribution (14).

In the current study, the prevalence of *Toxocara* infection in males (5.9%) was significantly higher than females (1.7%) suggesting gender classification as a partial risk factor partially related to differences in type of boys’ games and the specific behaviors of boys against *Toxocara* infection (15). Male gender was considered as a contributing factor (15). On the contrary, studies in Arak, Shiraz, Nigeria, and China showed higher prevalence among females (7, 8). The gender differences observed in different studies can be due to gender-specific behavior and type of games (7, 8).

In this study, although the infection was higher in the age range of 6–10 yr, age had no role in the prevalence of infection (12). However, significant dominance in the age groups of 1–10 yr was showed (16, 17). In this study, the mean age of positive cases was 8 yr. Seroepidemiologic studies on *Toxocara* showed that the children under 10 are at greater risk than other age groups. The higher incidence of this infection in younger children can be attributed to factors such as childish games and more contact with soil (18).

The infection rate was higher in urban areas than rural areas and the frequency of antibody was higher in rural areas (4.4%) than urban areas (1.6%) (19).The results of this study were similar to a study in Zanjan but showed less prevalence than the study in Shiraz (19, 20). In addition, the prevalence of infection was also assessed in the tribal region that showed a higher prevalence than urban and rural residents did. Regarding the fact, that prevalence of *Toxocara* infection was significantly higher in the tribal areas than urban and rural areas, nomads residents are at greater risk of incidence of *T. canis* infection. The cultural habits and climate conditions can affect the high prevalence of the infection, which can be effective in higher rates of infection in tribes.

Dogs, as the primary host and the main vector of *Toxocara* parasite, play an important role in the transmission of infection to humans. Everybody was in contact with dogs, although their infection frequency was not prominent, included 1/3 cases. Higher frequencies were reported of *Toxocara* infection among people exposed to dogs (9%) and (23%), although none of these differences were statistically significant (18, 19). Besides, no significant association was found between these two factors (14). In comparison, in Brazil, contact with dogs has been proposed as a risk factor for *Toxocara* infection (21). Generally, the transmission of infection due to contact with dogs can be due to the conditions of keeping animals, such as food, place, and defecation sanitation of animals affected the *Toxocara* infection in the current and similar studies (12).

In our study, a significant association was observed between decrease in red blood cells and *T. canis* titration might indicate a chronic disease (22). Laboratory changes, especially changes in the level of blood eosinophils, are reported as one of the most common signals of parasitic infection. The highest frequency of positive antibody among patients with high eosinophil was 5%. In positive antibodies against *T. canis*, the highest frequency was observed in 6%–10% of eosinophilia cases (1.8%), while the serological prevalence of infection among individuals with eosinophil of 1–5% and over 10% were zero and 2.3%, respectively. Significant association was found between prevalence of infection and high percentage of eosinophils. The prevalence of infection was significantly associated with high eosinophil levels, while another research did not find such an association (13, 15, 23). This association can be explained as the following: *T. canis* larvae secrete large amounts of glycosylated proteins that causes production of immunoglobulin E in blood samples of children, as prolonged exposure to the parasite shows chronic infection (increased immunoglobulin G) (24).

In our study, no association was found between infection and the level of parents’ education (7), while it was significant in study reported that lower educational level and more contact with infected sources like dog and soil are the main risk factors of *Toxocara* infection (25).

The prevalence of *T. canis* in dogs was about 27%. In addition, *Toxocara* eggs were seen in 8 out of 30 urban dogs (26%) and 3 out of 10 rural dogs (30%). The higher infection of rural dogs can be related to climatic conditions and the type of animal’s food in this region. *Toxocara* infection in cats and dogs in Iran has been reported in average around 26% (7). Compared to the prevalence obtained in this study, the rate of *Toxocara* infection in dogs was similar to the mean rate in the country. In another study, frequency of *T. canis* in studied dogs in Northern Iran was estimated as 60% (22) that was higher than the prevalence found in this study. Since dogs are important vectors in the transmission of *Toxocara*, checking the infection in dogs of the province is recommended. Controlling the dog population should be considered in residential areas.

## Conclusion

*Toxocara* infection in Sistan and Baluchestan, Iran, especially in the north (Sistan) was less than many other parts of the country, while the risks of infection among dogs are similar to other regions. Larger studies in the whole province, especially among tribes, are recommended for more accurate determining the prevalence of this infection.

## References

[1] Abdi J Darabi M Sayehmiri K Epidemiological situation of toxocariasis in Iran: meta-analysis and systematic review. Pak J Biol Sci. 2012; 15 (22): 1052– 5. 24261119 10.3923/pjbs.2012.1052.1055

[2] Boschetti A Kasznica J Visceral larva migrans induced eosinophilic cardiac pseudotumor: a cause of sudden death in a child. J Forensic Sci. 1995; 40 (6): 1097– 9. 8522917

[3] Kwon N-H Oh M-J Lee S-P Lee B-J Choi D-C The prevalence and diagnostic value of toxocariasis in unknown eosinophilia. Ann Hematol. 2006; 85 (4): 233– 8. 16463154 10.1007/s00277-005-0069-x

[4] Avcioglu H Burgu A Seasonal prevalence of *Toxocara* ova in soil samples from public parks in Ankara, Turkey. Vector Borne Zoonotic Dis. 2008; 8 (3): 345– 50. 18494602 10.1089/vbz.2007.0212

[5] Bede O Szénási Z Danka J Gyurkovits K Nagy D Toxocariasis associated with chronic cough in childhood: a longitudinal study in Hungary. J Helminthol. 2008; 82 (4): 357– 63. 18752712 10.1017/S0022149X0804827X

[6] Coelho LM Silva MV Dini CY Giacon Neto AA Novo NF Silveira EP Human toxocariasis: a seroepidemiological survey in schoolchildren of Sorocaba, Brazil. Mem Inst Oswaldo Cruz. 2004; 99 (6): 553– 7. 15558160

[7] Mosayebi M Hajihossein R Didehdar M Eslamirad Z Ejtehadifar M Hamzelo Z The role of *Toxocara* larva migrans in hypereosinophilia with unknown origin in patients referred to laboratories. Journal of Kermanshah University of Medical Sciences. 2014; 18 (3): 173– 180.

[8] Fallah M Azimi A Taherkhani H Seroprevalence of toxocariasis in children aged 1–9 years in western Islamic Republic of Iran, 2003. East Mediterr Health J. 2007; 13 (5): 1073– 7. 18290400 10.26719/2007.13.5.1073

[9] Ebrahimi Fard SF Fakhar M Sharif M Shirvani N Davood S Seroprevalence of *Toxocara* Infection among Adult Individuals with Eosinophilia in Babol, 2013. J Mazandaran Univ Med Sci. 2015; 24 (121) 355– 362.

[10] Roy MS India and Iran Relations: Sustaining the Momentum. ISDA issue brief, Institute for Defense Studies and Analyses. 2013; 56: 599– 617.

[11] Ghaderi A Abduli M Karbassi A Nasrabadi T Khajeh M Evaluating the Effects of Fertilizers on Bioavailable Metallic Pollution of soils, Case study of Sistan farms, Iran. Int J Environ Res. 2012; 6 (2): 565– 70.

[12] Fallah E Abdolrasool M Nasrin H Mohami Oskouei L The effect of dogs and cats at risk of toxocariasis. Yafte J Med Sci. 2012; 13 (4): 65– 71.

[13] Fillaux J Magnaval J-F Laboratory diagnosis of human toxocariasis. Vet Parasitol. 2013; 193 (4): 327– 36. 23318165 10.1016/j.vetpar.2012.12.028

[14] Maraghi S Rafiei A Hajihossein R Sadjjadi S Seroprevalence of toxocariasis in hypereosinophilic individuals in Ahwaz, southwestern Iran. J Helminthol. 2012; 86 (2): 241– 4. 21733275 10.1017/S0022149X11000307

[15] Baboolal S Rawlins SC Seroprevalence of toxocariasis in schoolchildren in Trinidad. Trans R Soc Trop Med Hyg. 2002; 96 (2): 139– 43. 12055800 10.1016/s0035-9203(02)90281-6

[16] Doğan N Dinleyici Eç Bor Ö Töz Sö Özbel Y Seroepidemiological survey for *Toxocara canis* infection in the northwestern part of Turkey. Turkiye Parazitol Derg. 2007; 31 (4): 288– 91. 18224618

[17] Espinoza YA Huapaya PE Roldán WH Jiménez S Abanto EP Rojas CA Seroprevalence of human toxocariasis in Andean communities from the Northeast of Lima, Peru. Rev Inst Med Trop São Paulo. 2010; 52 (1): 31– 6. 20305952 10.1590/s0036-46652010000100005

[18] Akhlaghi L Ourmazdi H Sarafnia A Vaziri S Jadidian K Leghaii Z An Investigation on the Toxocariasis Seroprevalence in Children (2–12 Years Old) from Mahidasht Area of Kermanshah Province (2003–2004). Razi J Med Sci. 2006; 13 (52): 41– 8.

[19] Nourian A Amiri M Ataeian A Haniloo A Mosavinasab S Badali H Seroepidemiological study for toxocariasis among children in Zanjan-northwest of Iran. Pak J Biol Sci. 2008; 11 (14): 1844– 7. 18817228 10.3923/pjbs.2008.1844.1847

[20] Sadjjadi S Khosravi M Mehrabani D Oryan A Seroprevalence of *Toxocara* infection in school children in Shiraz, Southern Iran. J Trop Pediatr. 2000; 46 (6): 327– 30. 11191141 10.1093/tropej/46.6.327

[21] Mendonca LR Figueiredo CA Esquivel R Fiaccone RL Pontes-de-Carvalho L Cooper P Seroprevalence and risk factors for *Toxocara* infection in children from an urban large setting in Northeast Brazil. Acta trop. 2013; 128 (1): 90– 5. 23845771 10.1016/j.actatropica.2013.06.018 PMC7612831

[22] Daryani A Sharif M Amouei A Gholami S Prevalence of *Toxocara canis* in stray dogs, northern Iran. Pak J Biol Sci. 2009; 12 (14): 1031– 5. 19947182 10.3923/pjbs.2009.1031.1035

[23] Sarkari B Lari M Shafiei R Sadjjadi SM A Comparative Seroprevalence Study of Toxocariasis in Hypereosinophilic and Apparently Healthy Individuals. Arch Pediatr Infect Dis. 2015; 3 (2): e17911.

[24] Rober MK Stanton Bonita F St. Geme Joseph W III Nelson Textbook of Pediatrics. 19th ed. Elsevier 2011: 1227– 9.

[25] Negri EC Santarém VA Rubinsky-Elefant G Giuffrida R Anti-*Toxocara* spp. antibodies in an adult healthy population: serosurvey and risk factors in Southeast Brazil. Asian Pac J Trop Biomed. 2013; 3 (3): 211– 6. 23620840 10.1016/S2221-1691(13)60052-0 PMC3631752

